# Heritability of *Apis mellifera* recapping behavior and suppressed mite reproduction as resistance traits towards *Varroa destructor*

**DOI:** 10.3389/finsc.2023.1135187

**Published:** 2023-02-21

**Authors:** Martin Gabel, Andreas Hoppe, Ricarda Scheiner, Jörg Obergfell, Ralph Büchler

**Affiliations:** ^1^ Landesbetrieb Landwirtschaft Hessen, Bieneninstitut Kirchhain, Kirchhain, Germany; ^2^ Universität Würzburg, Verhaltensphysiologie und Soziobiologi, Würzburg, Germany; ^3^ Länderinstitut für Bienenkunde Hohen Neuendorf e. V., Hohen Neuendorf, Germany; ^4^ Gemeinschaft der europäischen Buckfastimker e.V., Kassel, Germany

**Keywords:** breeding, selection, performance testing, genetics, heredity, honeybees

## Abstract

The selection of honeybee strains resistant to the ectoparasitic mite *Varroa destructor* is generally considered as one of the most sustainable ways of coping with this major bee parasite. Thus, breeding efforts increasingly focus on resistance parameters in addition to common beekeeping traits like honey yield and gentleness. In every breeding effort, the success strongly depends on the quantifiability and heritability of the traits accounted. To find the most suitable traits among the manifold variants to assess *Varroa* resistance, it is necessary to evaluate how easily a trait can be measured (i.e., testing effort) in relation to the underlying heritability (i.e., expected transfer to the following generation). Various possible selection traits are described as beneficial for colony survival in the presence of *Varroa destructor* and therefore are measured in breeding stocks around the globe. Two of them in particular, suppressed mite reproduction (SMR, *sensu lato* any reproductive failure of mother mites) and recapping of already sealed brood cells have recently gained increasing attention among the breeders because they closely resemble resistance mechanisms of some *Varroa*-surviving honeybee populations. However, it was still unknown whether the genetic background of the trait is sufficient for targeted selection. We therefore investigated the heritabilities and genetic correlations for SMR and REC, distinguishing between recapping of infested cells (RECinf) and all cells (RECall), on an extensive dataset of Buckfast and Carniolan stock in Germany. With an accessible h² of 0.18 and 0.44 for SMR and an accessible h² of 0.44 and 0.40 for RECinf, both traits turned out to be very promising for further selection in the Buckfast and Carnica breeding population, respectively.

## Introduction

Modern breeding approaches in the honeybee realm are based on the needs of apiculture. The ectoparasitic mite *Varroa destructor* [Anderson & Trueman (Mesostigmata: Varroidae)] plays a key role for honeybee health by harming its host *Apis mellifera* [Linnaeus (Hymenoptera: Apidae)] through direct feeding and virus vectoring (1–3). Thus, the mite presents unambiguously a major task for both, apiculture (2, 3) and honeybee breeding efforts (4–6) worldwide. Currently, most colonies of *A. mellifera* managed for apiculture depend on regular miticide treatments to survive the *Varroa* infestation (2). Breeding towards resistance against this parasite seems to be the most promising and sustainable way of dealing with this problem (6, 7), although this approach does not offer an immediate solution for the global beekeeping industry (8). It rather represents a part of integrated pest management strategies to achieve a sustainable coexistence between mites and bees under beekeeping conditions (5, 9, 10).

Resistance can be scored in a broader sense in a) surviving and b) not surviving the mite infestation without treatment. Based on the fundamental idea of natural selection, this dichotomous approach has been used successfully in some untreated breeding populations (11–13). The general idea of natural selection thereby arose from the survival of mostly free-living and unmanaged colonies described in several locations around the world (12, 14–19). This selection approach proofed to be a valuable strategy, in parts acting as a role model for many breeding efforts (5, 20) and biotechnical methods of *Varroa* control (9). Under central European conditions however, it is difficult to implement this system strictly (i.e., colonies either survive or die) in larger breeding programs or management practices. Although the intensity and structure of breeding schemes differs clearly between countries, the different programs typically focus on additional desirable beekeeping traits besides *Varroa* resistance measures (5, 21–25). Thus, a more detailed resistance scoring scale is needed for comparisons among colonies already selected for other beekeeping traits. To achieve such comparability in *Varroa* resistance, selective breeding has been applied to several scorable traits which are beneficial to the health of honeybee colonies. This includes a) the proportion of mites removed by grooming, b) the share of removed injured brood cells or c) the post capping duration of broodcells (see reviews by 5, 10, 26), among others. In contrast to the mere survival of colonies, this approach aims to select the underlying mechanisms of resistance, which were found to play key roles in naturally surviving populations (14, 26, 27). Since the effects of different resistance traits are likely to sum up or act synergistically together (14), it seems reasonable to account for several resistance traits in parallel (28). Thus, various described survival mechanisms have been tested as possible selection criteria (6, 26, 28).

As for other beekeeping related traits, the selection progress is thereby highly dependent on organized breeding schemes (21), controlled mating (29) and most importantly, heritable traits (5, 8). If all of these basic requirements are met, the progress in selected traits can be substantial within a few generations (21). This applies especially when detailed knowledge on the heritability of traits is used to calculate breeding values as a guidance for selection decisions (21, 30, 31).

Mite population development (VID) and hygienic behavior are the most frequently tested traits related to *Varroa*. For both characters, significant selection effects were achieved through selective breeding in a big managed population (5, 21). Among the above-mentioned requirements, this selection progress based especially on the practicable and standardized testing protocols for both traits (21, 22). While the hygenic behavior turned out to be strongly hereditary anyway (h²= 0.52), the comparatively low heritability of VID (h²= 0.11) thereby seemed to be compensated by the simple testing procedure and thus extensive data base (21). On the other hand, several traits were discarded as selection criteria after a few generations, since their heritability proofed to be too low compared to the testing effort (reviewed in 5, 6, 10, 22, 26).

Three other candidate traits have frequently been associated with colony survival and are currently accounted for in breeding programs. These are a) *Varroa*-sensitive hygiene (VSH), b) suppressed mite reproduction (SMR, sensu lato) also called mite non-reproduction (MNR) according to (26), and c) the opening and recapping (REC) of already sealed brood cells (26, 27, 32, 33). As ruled out by Büchler et al. (5), any suitable selection trait needs to be both, heritable and easy to measure in practice. In case of VSH, a comparatively low heritability (h²) of 0.18 was described (8), while tedious measurements are required for data acquisition (34, 35). Nevertheless, it seems to be an important trait for colony survival (26, 36) and was successfully selected for in some commercially breeding lines (36). Interestingly, it also contributes strongly to reproductive failure of mites (i.e., SMR) on the long-term (26, 37), although the expression of this trait is also affected by other parameters (27, 32, 38, 39) and thus shows low repeatability in individual colonies (28, 40). Likewise, the REC behavior is commonly increased in surviving colonies (27, 32, 33) and can suppress the reproductive success of mites (27, 32). Compared to the measurement of VSH, the data acquisition on SMR and REC is rather simple, since an artificial infestation is not obligatory and sampled combs can be stored in the freezer up to brood investigation (41). Hence, both traits hold great potential for effective resistance breeding if the heritability would be high enough (40). To the best of the authors’ knowledge, heritability for SMR was only estimated once based on a dataset of 28 queens (42), while there is currently no estimation for the heritability of REC of *Varroa*-infested cells available. As pointed out by Eynard et al. (40), an large-scale estimation of heritability for SMR is urgently needed to use the potential of this trait more efficiently for resistance breeding. The same applies for REC of infested cells since this trait is gaining increasing attention in breeding efforts. Detailed knowledge on the heritability would also set the basis for breeding value estimation and thus enable a more targeted selection. Therefore, we estimated the heritability of SMR and REC based on an extensive dataset of Buckfast and Carniolan stock and implemented these traits in practical breeding value estimation schemes.

## Materials and methods

### Sources of measurement data

The majority of colonies (57% for Carnica, 100% for Buckfast) was tested between 2019 and 2021 in the framework of a nationwide selection program on suppressed mite reproduction (SMR) and recapping behavior (REC) in Germany [(43), **Table 1**]. In this project, several regional breeding groups and institutes jointly tested Buckfast and Carnica colonies for their expression of SMR and REC. Colonies were either full grown performance test colonies, or nuc-sized MiniPlus colonies. In the latter case, colonies were mostly headed by single-drone-inseminated queens (SDI) and partly by multi-drone-inseminated queens (MDI). Due to the limited egg laying capacity of the SDI queens, MiniPlus colonies were exclusively built up for brood investigations on REC and SMR. Full grown performance test colonies were additionally tested for common beekeeping traits (e.g., honey yield) according to the GdeB and AGT test protocols (24, 25). Within the framework of these test protocols (24, 25), selection decisions, e.g. mating choices, were made by the individual breeders.

**Table 1:** Number of colonies with brood investigations from the different data sources used.

Data source	Breeding Population	
	Carnica	Buckfast
**MiniPlus colonies** (43)	875	1,492
**Performance test colonies** (43)	632	292
**LLH and AGT** (28)	243	–
**Austria** (28)	147	–
**Croatia** (28)	135	–
**Netherlands** (44)	193	–
**LIB** (44)	89	–
**Other breeders in BeeBreed** (44)	216	–
**Total**	**2,634**	**1,784**

Abbreviations are given in the supplements.

**Figure 1:**
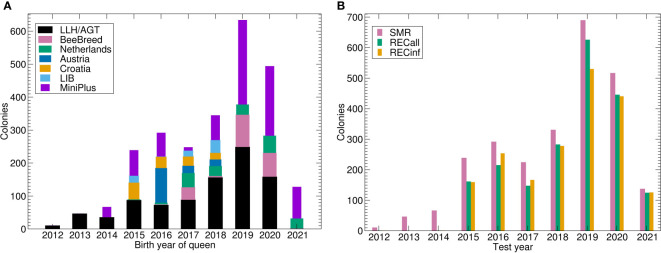
Number of colonies under brood investigation for the Carniolan breeding population. **(A)** Number of colonies tested by birth year of queen and data source. LLH/AGT comprises both performance tested colonies within (43) and previous projects of LLH and AGT. **(B)** Number of phenotypes by trait and test year. Note that the number of SMR is higher because REC was only recorded for a fraction of colonies. Abbreviations are given in the supplements.

**Figure 2:**
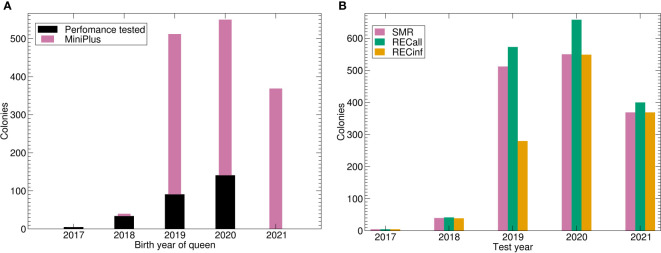
Number of colonies under brood investigation for the Buckfast breeding population. **(A)** Number of colonies tested by birth year of queen and data source. **(B)** Number of phenotypes by trait and test year. Abbreviations are given in the supplements.

Another data source was the predecessor project at LLH Bee Institute Kirchhain (LLH), which comprised colonies from Austria and Croatia (28). Other measurements have been deposited to BeeBreed (44) in two projects at Länderinstitut für Bienenkunde e.V. (LIB), by Dutch breeders and further breeders unrelated to the previously mentioned projects (**Table 1**, **Figure 1**). See **Figure 2** for a graphical representation of the number of Buckfast colonies.

### Brood investigations on suppressed mite reproduction (SMR) and recapping behavior (REC)

Brood combs were either investigated immediately after sampling (i.e., alive), or stored at -20°C until investigation. The occurrence of SMR was investigated in single infested cells. The expression of REC was assessed over all cells (RECall) and infested cells (RECinf) separately. Therefore, brood cells were opened separately to investigate the underside of the cell cap for signs of recapping, i.e. holes in the pupal cocoon. Afterwards, cell infestation and reproductive status of mites was examined. All investigations followed the protocol of the Research Network on Sustainable Bee Breeding (39, 41). Accordingly, reproductive failure in terms of SMR was defined as a single infested cell containing either a) no mite offspring (i.e., infertile), b) only female offspring (i.e., male missing) or c) mite offspring, which was too young in comparison to the developmental stage of the respective host bee pupae (i.e., delayed reproduction).

For the majority of full-grown colonies, up to 1000 brood cells were opened until 25 single infested cells were found (43). For MiniPlus colonies, up to 300 brood cells were opened until at least 10 single infested cells were found (43). In heavily infested colonies, more infested brood cells were analyzed. For SMR and RECinf calculations in (43), values obtained from less than 10 single infested cells were discarded. For some colonies analyzed by external contributors, other standards might have been applied.

For Carnica colonies, the clearance rate in pin tests has been obtained following the AGT protocol (25).

### Pedigree information

Respective pedigree information for each queen were derived from the Beebreed-Database (44) for Carniolan stock or the Pedigree-Database (45) for Buckfast stock, respectively. For the calculation of genetic parameters, a sub-pedigree was created which contained all colonies investigated and their complete ancestor trees. Thus, for Carnica 3.250 colonies and for Buckfast 2.592 colonies were added to complete the pedigree.

### Breeding model

Models of SMR, RECall and RECinf have been set up as mixed-linear models with a direct genetic effect (effect of the worker community), a maternal genetic effect (effect of the queen), a fixed effect of the investigation series and country (MiniPlus, LLH/AGT, Austria, Croatia, Netherlands, LIB, other for Carnica; MiniPlus and performance tested colony for Buckfast) and a random effect, analogous to (21). As the colonies have not been organised in comparative testing apiaries (some breeders sent in brood samples of only one colony per apiary), a fixed apiary effect was out of the question.

### Parameter estimation

The genetic parameters (i.e., heritabilities and genetic correlations) have been calculated with programs of the BLUPF90 series (46) in an iterative process, as follows.

First, AIREML was run. If AIREML converged and the parameters were not on the boundary (either the genetic variance vanished, or the residual variance vanished), the result was verified with a subsequent REML run. If AIREML was not successful, different start parameters were used. If AIREML was still not successful with these parameters, REML was run. The results of REML were then used in a subsequent AIREML run to confirm the result and obtain the standard errors of the parameters.

Initially, single-trait models were run to estimate the genetic variance, the genetic covariance between the direct and maternal effect and the residual variance. Then, two-trait models were composed of each combination of one-trait models with the final parameters.

From genetic and residual variances for both direct and maternal effects, the workers’ effect heritability was calculated as ${\text{h}}_{w}^{2}=\sigma_{\text{AW}}^{2}/\sigma_{\text{AP}}^{2}$ , where $\sigma_{\text{AW}}^{2}$ is the additive variance of the workers’ effect and $\sigma_{\text{AP}}^{2}$ the phenotypical variance calculated as $\sigma_{\text{AP}}^{2}={\text{a}}_{\text{ss}}\sigma_{\text{AW}}^{2}+\sigma_{\text{AQ}}^{2}+\sigma_{\text{AQAW}}+\sigma_{\text{E}}$ , where a_SS_ is the additive genetic relationship between two drone producing queens reared from the same colony, $\sigma_{\text{AQ}}^{2}$ is the additive variance of the queen effect, σ_AQAW_ is the genetic covariance of queen’s and workers’ effect and σ_E_ is the residual variance. The queen’s effect heritability was calculated as ${\text{h}}_{Q}^{2}=\sigma_{\text{AQ}}^{2}/\sigma_{\text{AP}}^{2}$ , the heritability of the selection criterion was calculated as ${\text{h}}_{\text{SC}}^{2}=(\sigma_{\text{AQ}}^{2}+\sigma_{\text{AW}}^{2}+2\sigma_{\text{AQAW}})/\sigma_{\text{AP}}^{2}$ . The accessible heritability was calculated as ${\text{h}}_{A}^{2}={\text{a}}_{\text{ss}}{\text{h}}_{\text{SC}}^{2}$ . See Hoppe et al., 2020 (21) for more details.

### Breeding values estimation and validation

For the breeding values estimation, the pedigree was extended to contain siblings (and their siblings, iteratively) of colonies. Additionally, a pedigree entry for the colony was added, which represents the expectation value of an offspring queen. To calculate the breeding values, BLUPF90 was used (46).

The genetic trend shows the yearly averages of all breeding values per year obtained from colonies with measured SMR phenotypes.

To estimate the predictive power of the breeding model, the following validation procedure was used which relates the breeding values calculated ignoring the phenotypes of the test year (and all subsequent years) with the phenotypes of the test year. The breeding values were estimated with the full pedigree, while the phenotypes were discarded. As a first measure, the Pearson correlation coefficients between the breeding values and the phenotypes (adjusted for the fixed effect) were calculated. As a second measure, all tested colonies of the test year were sorted by their breeding values and split into four quartiles. Then, the average phenotype (adjusted for the fixed effect) of each quartile was calculated. This process was iterated for test years 2017 to 2022 for the Carncia population and 2019 to 2022 for the Buckfast population.

## Results

### Carnica

The calculation of genetic parameters was feasible for all single-trait and double-trait models. All investigated traits show comparatively high heritabilities (**Table 2**).

**Table 2:** Heritabilities in Carnica colonies.

	Trait			
	SMR	RECall	RECinf	Pintest
**Accessible h^2^_A_**	0.44 ( ± 0.06)	0.46 ( ± 0.06)	0.40 ( ± 0.06)	0.72 ( ± 0.08)
**Selection Criterion h^2^_SC_**	0.82 ( ± 0.11)	0.86 ( ± 0.12)	0.76 ( ± 0.12)	1.36 ( ± 0.16)
**Queen h^2^_Q_**	0.57 ( ± 0.11)	0.48 ( ± 0.12)	0.30 ( ± 0.10)	0.47 ( ± 0.14)
**Worker h^2^_W_**	1.22 ( ± 0.10)	0.51 ( ± 0.09)	0.44 ( ± 0.09)	1.17 ( ± 0.16)
**Queen/Worker r_QW_**	–0.90 ( ± 0.03)	–0.44 ( ± 0.13)	–0.36 ( ± 0.20)	–0.64 ( ± 0.10)

For the calculation of the different types of heritabilities and correlations see (21). Standard errors are given in brackets. Abbreviations are given in the supplements.

The worker heritabilities ${\text{h}}_{w}^{2}$ for SMR and pintest show a peculiarity of heritabilities larger than 1. This is possible because “the worker” is not a single animal but a collection of individuals. However, this heritability is not accessible to selection because one can only use individual animals for selection and not the full community of workers. This effect is compensated for by the correction formula of the accessible selection (21).

The heritability is highest for pintest, which puts the potential for selection progress into perspective. The heritability is very similar for SMR and both recapping traits, regarding the standard error.

The genetic correlations between the queen and worker effect are strongly negative, especially for SMR (**Table 3**).

**Table 3:** Genetic correlations between traits in Carnica colonies.

Trait	RECall	RECinf	Pintest
SMR	*0.078* ( ± 0.12)	*-0.064* ( ± 0.12)	0.42 ( ± 0.11)
RECall		0.79 ( ± 0.06)	0.38 ( ± 0.10)
RECinf			0.45 ( ± 0.11)

Correlation coefficients are given in italics if the confidence interval (given by AIREML standard error of the correlation) contains zero, i.e., if the correlation is not significantly different from zero. Standard errors are given in brackets. Abbreviations are given in the supplements.

The genetic correlation (see **Table 3**) is highest between both recapping traits. SMR can be considered as not correlated to both recapping traits, the low values are overshadowed by the standard errors. Interestingly, the pintest is correlated to SMR and both recapping traits with a medium correlation coefficient, which presents a partly paradox finding.

**Figure 3:**
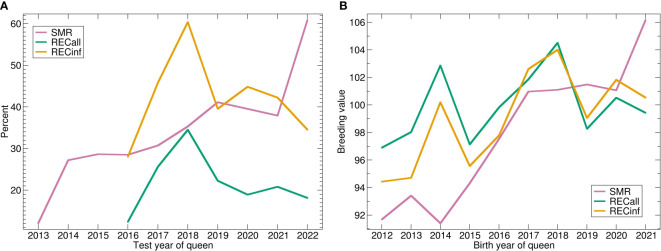
**(A)** Phenotypic and **(B)** genetic trends for SMR, RECall and RECinf in the Carniolan population. Abbreviations are given in the supplements.

The trend of the phenotypes (**Figure 3A**) shows a strong upward trend for SMR, while REC starts low, peaks in 2018 and decreases again. The genetic trend (**Figure 3B**) shows a similar picture. For SMR, there is a strong genetic trend upwards. Apparently, the stock was successfully selected for SMR. The genetic effect of both recapping traits starts lower than the current level, respectively. The trend is very similar for both Recapping traits, not surprisingly because of the high genetic correlation. In comparison between the two Recapping traits, the genetic trend is stronger for RECinf.

**Figure 4:**
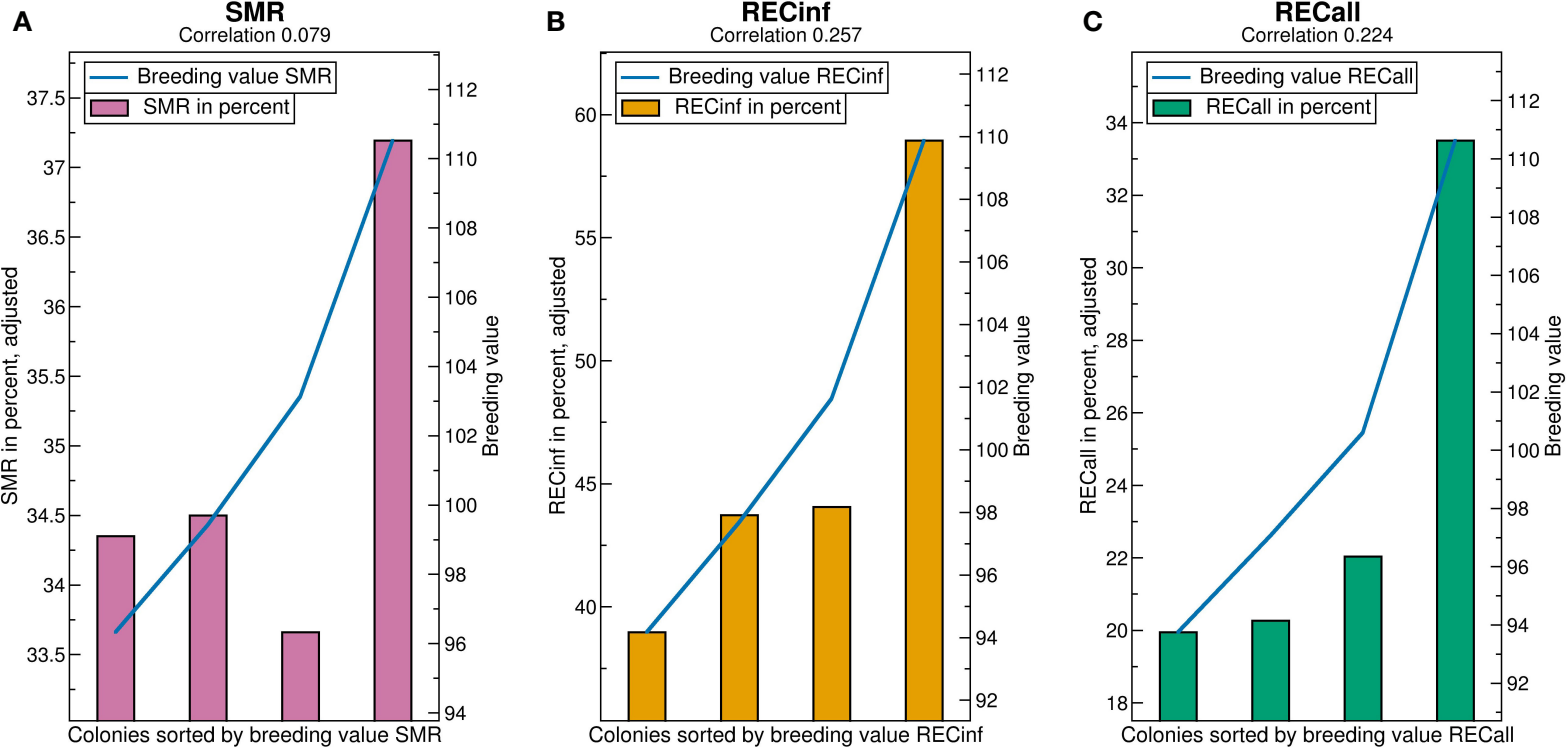
Validation charts for **(A)** SMR, **(B)** RECinf and **(C)** RECall breeding values in the Carnica population. Abbreviations are given in the supplements.

See **Figure 4** for the breeding value validation charts. Comparing the correlations, i.e., predictivity of breeding values, it is best for RECinf and also very good for RECall, but comparatively poor for SMR. For all traits, the best quartile has by far the highest phenotypes, whereas the lower quartiles do not show large differences, i.e., the higher breeding values are the more predictive. The y-axis-scales reveal that the phenotypical differences between the quartiles are very high for RECinf, high for RECall and low for SMR. The difference between the highest and lowest quartile for SMR is less than 3 percentage (**Figure 4A**).

The high predicitvity of the recapping traits shows that it would be possible to effectively select for RECinf and RECall, but apparently, this has not been done in the investigated population. For RECinf, the highest quartile is 20 percent points higher than the lowest (**Figure 4B**). For RECall, the highest quartile is by 14 percent points larger than the other three (**Figure 4C**).

### Buckfast

The calculation of genetic parameters for the Buckfast population was feasible for all traits including the two-trait models. This is a remarkable result because the number of phenotypes was considerably smaller than for the Carnica population and it does not span over generations (**Table 1**; **Figure 2A**).

The heritabilities for SMR and RECall in the Buckfast population are smaller than in the Carnica population, while for RECinf they are slightly higher (see **Table 4**). All traits are dominated by the worker effect.

**Table 4:** Heritabilities in Buckfast colonies.

	Trait		
	SMR	RECall	RECinf
**Accessible h** ^2^_A_	0.18 ( ± 0.07)	0.33 ( ± 0.07)	0.44 ( ± 0.09)
**Selection Criterion h** ^2^_SC_	0.34 ( ± 0.14)	0.62 ( ± 0.13)	0.83 ( ± 0.18)
**Queen h** ^2^_Q_	0.25 ( ± 0.13)	0.12 ( ± 0.08)	0.16 ( ± 0.12)
**Worker h** ^2^_w_	0.32 ( ± 0.11)	0.22 ( ± 0.09)	0.32 ( ± 0.12)
**Queen/Worker r**_QW_	–0.66 ( ± 0.79)	*0.21 ( ± 1.9)*	*0.12 ( ± 1.11)*

For the calculation of the different types of heritabilities and correlations (21). Standard errors are given in brackets. Abbreviations are given in the supplements.

The genetic correlation between queen and worker effect is negative for SMR, similar to the parameters in the Carnica population. For both recapping traits the genetic correlation between queen and worker effect is positive. However, the standard errors are so large that it is not considered significantly distinguishable from zero.

The genetic correlation between both recapping traits is even higher than in the Carnica population (**Table 5**). It is so close to one that it suggests it may be not possible to select for RECinf without also increasing RECall. The correlation from SMR to RECall is slightly negative regarding the standard error at the same amount. The genetic correlation of SMR to RECinf is effectively zero at this level of standard error.

**Table 5:** Genetic correlations between traits in Buckfast colonies.

Trait	RECall	RECinf
SMR	*-0.18* ( ± 0.18)	*-0.026* ( ± 0.27)
RECall		0.96 ( ± 0.02)

Correlation coefficients are given in italics if the confidence interval (given by AIREML standard error of the correlation) contains zero, i.e., if the correlation is not significantly different from zero. Standard errors are given in brackets. Abbreviations are given in the supplements.

**Figure 5:**
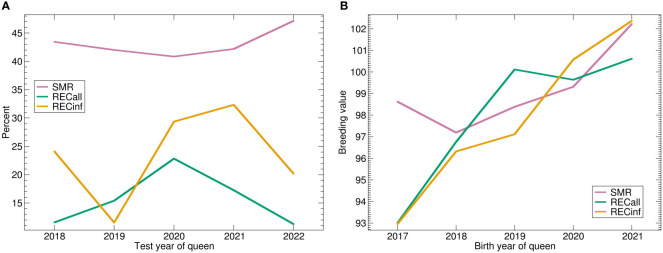
**(A)** Phenotypic and **(B)** genetic trends for SMR, RECall and RECinf in the Buckfast population. Abbreviations are given in the supplements.

Although only 5 years are represented, a positive genetic trend is visible for all traits (**Figure 5**). These trends are more apparent in the genetic trends than in the phenotypes where they are nearly invisible. The genetic trend is much stronger for RECinf and RECall than for SMR.

**Figure 6:**
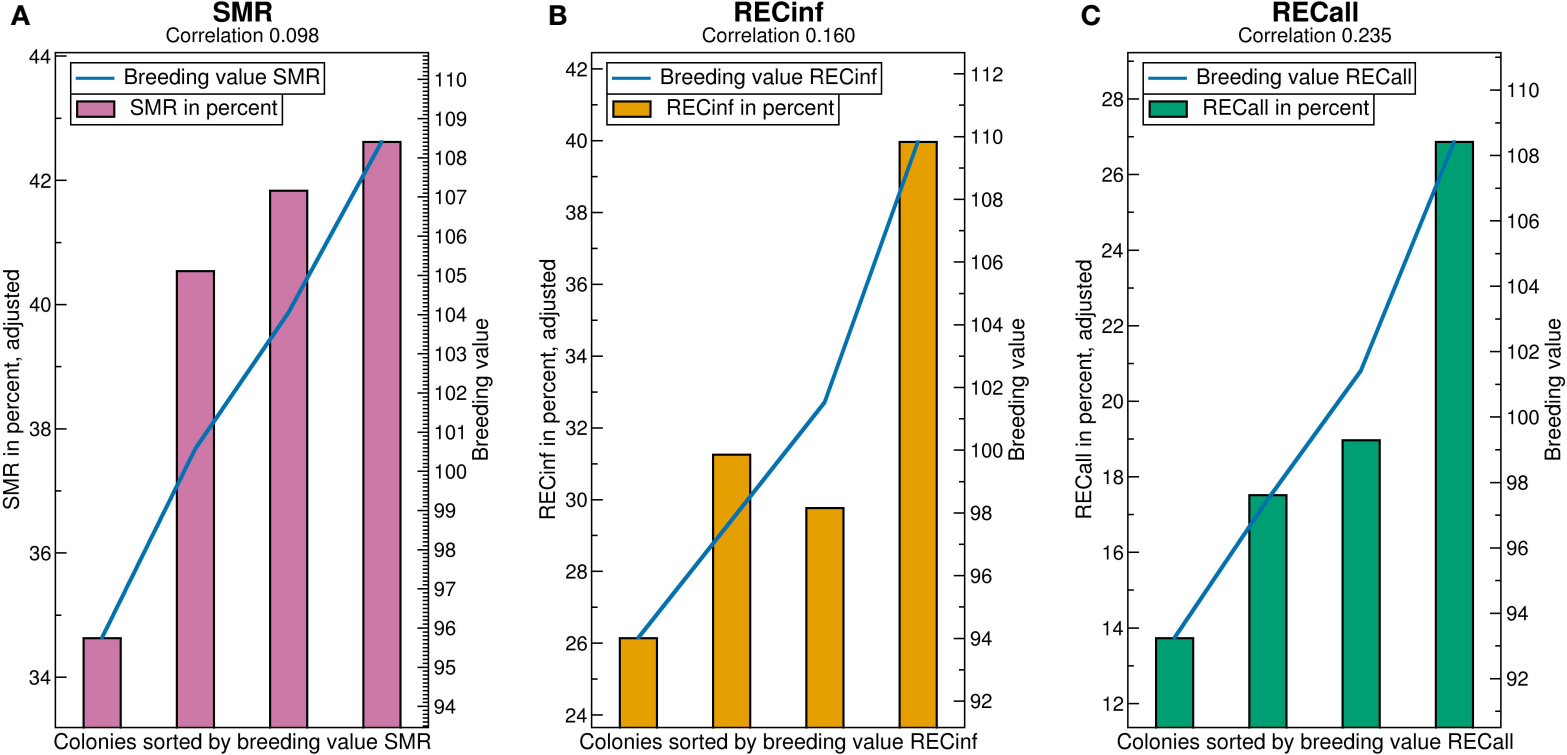
Validation charts for **(A)** SMR, **(B)** RECinf and **(C)** RECall breeding values in the Buckfast population. Abbreviations are given in the supplements.

The predictivity of breeding values (correlations in **Figure 6**) is highest for RECall (0.235), followed by RECinf (0.160), and lowest for SMR (0.098). In comparison to the results in the Carnica population, the ranking among the traits RECall and RECinf is reversed. The predictivity of SMR and RECall is somewhat higher than in the Carnica population, whereas the predictivity for RECinf is much lower.

For SMR, the low quartile separates from the rest, as opposed to the Carnica population (**Figure 4A**) where the highest quartile stands out. The difference between the highest and lowest quartile is about 8 percent points higher than in the Carnica population. For RECall and RECinf, the result resembles the results found in the Carnica population, where the highest quartile is considerably higher than the others. The difference between the highest and lowest quartile is about 14 percent points for both.

## Discussion

We have demonstrated that it is possible to estimate genetic parameters for SMR, RECinf and RECall and that the derived breeding models are valid despite relatively few assessed generations. This sets a valuable yardstick on how many colonies are necessary to understand the genetic properties of new traits. In addition, the heritabilities of all traits are relatively large showing good selection potential in both populations. However, the predictivity of breeding values for SMR is quite low, which is in concordance with the reported low repeatability of SMR measurements (28). This results in an interesting paradox, that although measuring SMR is comparatively inaccurate, selection for this trait is still effective. Similar results have been shown in the BeeBreed Carnica population selected for low VID (21). Similar to the values presented for SMR in the present study, the breeding values for this trait hold a very low predictivity of 0.081 (21). Despite this, breeding value based selection resulted in substantial genetic progress for VID (21). Thus, regardless of their low predictivity, the selection based on breeding values rather than raw measurements of phenotypes will most probably also advance the selection on SMR.

Hence, we have shown that SMR, RECinf and RECall can be increased by targeted selection, and indeed have increased already in few breeding generations. However, it must be noted that this alone does not guarantee complete resistance to *Varroa*, and finally improved colony vitality and decreased necessity of *Varroa* management practices in the future. Resistance *per se* is a varying combination of several possible traits acting together in the respective environment (5, 47) thereby reducing the reproductive ability of the parasite (26). In contrast to easily measurable traits like honey yield, the generic term *Varroa* resistance is therefore difficult to quantify in an accuracy required for selective breeding on a larger scale, where typically also other selection parameters are accounted for (5, 21–25, 45). By adapting measurable traits like SMR and REC from surviving populations (14, 26, 27, 32, 33) to bigger managed populations under targeted selection, we aim to include more aspects of resistance into locally adapted and selected stocks. In case of SMR and REC, average values of 45% SMR and 55% RECinf were recently reported for surviving populations (reviewed by (33)) which seems reachable for both breeding populations investigated in the present study. However, it is very important to avoid a situation in which breeds show high values of SMR and REC but are less adapted to other factors or are unmanageable for beekeeping. The estimation of genetic correlations between SMR, RECinf and RECall on the one hand and the traditional breeding traits on the other hand serves to foresee problems of this kind. Therefore, a continuation of brood investigation as part of the performance test is highly recommended, since such information cannot be obtained from MiniPlus colonies.

In addition, it seems to be important to account for multiple resistance traits in the future. Besides their possibly varying importance discussed above, several of these traits appear to be linked and thus selected in parallel. Despite the fact that only SMR was measured in the beginning, the genetic trend for both REC traits also started lower than the current level. This indicates that the genetic progress obtained for SMR also unintentionally led to an increase in REC as well, before REC parameters were even accounted. Likewise, the small range of SMR breeding values in the Carnica population indicates that the strong genetic trend for this trait is partly dependent on the selection of other causally linked parameters, e.g., low *Varroa* infestation development (VID).

Also, the traditional breeding traits do not fully represent what is needed to assess vitality in the context of *Varroa* burden. It is therefore recommended to transform vitality scores already quantified in scientific studies (e.g (48).,) into regular breeding traits. As a very accurate approach in this direction, the AGT recommends the so-called “vitality test”, a protocol to postpone *Varroa* treatment until a critical infestation is reached, which implies constant monitoring of the infestation level (25). However, up to now this serves mainly as an additional information for the breeders. What is lacking is an outcome variable of this “vitality test” both readily applicable to the regular breeder and expressive for the mathematical model. Here, more research is necessary.

As the Carnica population considered in this study overlaps with the BeeBreed Carnica population (21) and most performance test colonies and even some MiniPlus colonies underwent pintest, a comparison with the genetic parameters of the pintest can be made. The heritability obtained in the present study (h²= 0.72) is much higher than in the BeeBreed Carnica population (h²= 0.21) (21). To explain this difference, it has to be noted that there is a fundamental difference in the genetic model applied for the pintest in these calculations. In the Carnica breeding system, a fixed effect of testing apiary and year is applied. Thus, environmental effects of the test season are removed from the breeding values. This results in a high predictivity of the respective breeding values. Here, such a fixed effect could not be applied, because the brood samples were often derived from only one colony or very few colonies per testing apiary. It is known that a different definition of a fixed effect leads to very different heritability estimations. As the breeding values estimation with apiary-year fixed effects is appropriate, indicated by steep selection progress and high predictivity of breeding values (21), we conclude that the heritability for pintest estimated in the present study is artificially bloated. Thus, we may also assume that the heritabilities of SMR and recapping traits are bloated to the same extend. Consequently, it can be hypothesized that if SMR and REC were tested in an apiary-year context, its heritability would be likewise lower and thus approximately at the level of the honey yield heritability in the Carnica population (h²= 0.14) (21). However, this would absolutely suffice as the selection for honey yield, especially based on breeding values, has been proven to be very effective in practice (21). In fact, the negative genetic correlations between queen and worker effects for REC and SMR in the Carnica population investigated in the present study already indicate previous selection on these parameters. This is especially apparent in the strong genetic progress of SMR, while the quartile distribution for RECinf suggests that the potential for a bigger selection effect rather could be used in the future.

Despite this promising genetic background, SMR and REC measurements can be affected by various external factors (27, 32, 38, 39, 49). It thus seems to be rational to include apiary-year information in the raw data acquisition and breeding value estimation for following breeding efforts in order to increase the breeding value predictivity. In addition to apiary effects, it should be likewise accounted for variation through differences in data acquisition. Since the brood investigation methods require training and experience, the practical knowledge of investigators is likely to contribute to the variation of phenotype values. This might particularly apply for the investigation of MiniPlus colonies with SDI or MDI queens. These colonies are mostly investigated in smaller batches by private breeder groups, while samples from performance tested colonies are mostly processed by research institutions and professional investigators. Since the work of private breeders is essential for a broad genetic basis, while the additional work load of brood investigations in the season is immense, possible simplifications of the testing protocols need to be investigated. For instance, it is much easier to just score RECall (50, 51) and the question remains if, for a fraction of a population, this would be worthwhile. Again, this is linked to the estimation of genetic correlations between different traits. Another option would be brood investigation services offered by companies which evaluate SMR and REC in brood combs sent in by breeders. Similar services from professional investigators are common for morphometric analysis in the Carnica Population. Such a central evaluation of brood combs by trained investigators would not only ease the testing efforts for breeders, but also increase the accuracy of measurements. However, the question how the costs are shared among private breeders, breeding associations or the whole beekeeping community must be taken into account. Besides the promising genetic parameters of SMR and REC shown in the present study, such implementations in practicable performance testing procedures are urgently needed for successful selection. Without easy-to-apply test protocols for the practice, even heritable traits are unlikely to gain substantial genetic progress on the long-run. For example, breeding efforts for increased grooming behavior of the workers have largely stalled in Europe. Although some selection progress could be achieved in focussed breeding programs (20, 52), the heritability (h²) of 0.16 (53) or even lower (54) seemed to be insufficient for an ongoing large scale selection with laborious testing requirements (5, 52, 55). In case of SMR and REC, both investigated populations show promising heritabilities and genetic trends for these traits, but are likewise dependent on a large-scale performance testing of colonies.

To our surprise, the calculation of genetic parameters for the Buckfast population proved less problematic than expected from the fact that only three seasons of data recording could be used and values mainly derived from MiniPlus colonies. In addition, the concept of Buckfast breeding distinctively differs in some points from the methods widely used in Carnica breeding which may lead to differences in population structure. For example, selection is solely based on performance of colonies without any form of morphometric analysis of workers and drones. This also includes the performance testing of new and mainly unselected strains derived from different subspecies of *Apis mellifera*, beside the regular testing and selection of established Buckfast lines. This might partly explain differences in the genetic correlations between queen and worker effects for REC parameters, which were negative for Carnica colonies but positive for Buckfast colonies. Normally, positive correlations indicate a situation where the trait has not been selected previously but might be accessible for targeted selection. However, given the large standard errors, this is not guaranteed in this dataset. According to the quartile distribution for SMR in both populations, the selection for high SMR values can be predicted to be more effective in the Buckfast population (lowest quartile stands out) when compared to the Carnica population (highest quartile stands out). For RECinf, this trend seems to be inverse with less effective selection for high RECinf values in the Buckfast population.

In addition, a positive genetic trend was also visible for all traits in the Buckfast population. Interestingly, these trends were not apparent in the phenotypes, which shows that selection can occur without immediately being visible in the raw phenotype data.

To our knowledge, this is the first application of breeding value estimation in Buckfast stock. In the tradition of Brother Adam, Buckfast breeding relies on the validity of the direct (phenotypic) evaluation of colonies (56) and for over a century of successful breeding has not perceived the need for any form of breeding values. However, our study showed that the basic requirements for breeding value estimation, such as a meticulous recording of ancestry (45), are more than fulfilled for the Buckfast stock investigated in the present study.

## Data availability statement

The raw data supporting the conclusions of this article will be made available by the authors, without undue reservation.

## Author contributions

The investigation was designed by AH, MG, and RB. Data acquisition related to (43) was performed by JO and MG, other data sources listed in **Table 1** were handled by AH. Statistical analysis and calculation of genetic parameters was performed by AH. AH and MG prepared the first draft of the manuscript. In the following, RS, RB, JO, AH, and MG commented on earlier versions of the manuscript and contributed in the writing process. All authors contributed to the article and approved the submitted version.
